# The Centrality of Obesity in the Course of Severe COVID-19

**DOI:** 10.3389/fendo.2021.620566

**Published:** 2021-03-11

**Authors:** Danfei Liu, Tongyue Zhang, Yijun Wang, Limin Xia

**Affiliations:** Department of Gastroenterology, Institute of Liver and Gastrointestinal Diseases, Hubei Key Laboratory of Hepato-Pancreato-Biliary Diseases, Tongji Hospital of Tongji Medical College, Huazhong University of Science and Technology, Wuhan, China

**Keywords:** COVID-19, obesity, obesity-associated comorbidities, pathophysiology, therapeutic strategies

## Abstract

The ongoing coronavirus disease 2019 (COVID-19) pandemic caused by the novel severe acute respiratory syndrome coronavirus 2 (SARS-CoV-2) has become a global public health challenge. Most patients do not experience severe complications, but approximately 25% of patients progress to acute respiratory distress syndrome (ARDS), and the mortality rate is approximately 5–7%. Clinical findings have determined several risk factors for severe complications and mortality in COVID-19 patients, such as advanced age, smoking, obesity, and chronic diseases. Obesity is a common and serious health problem worldwide that initiates a cascade of disorders, including hypertension, cardiovascular disease (CVD), diabetes mellitus, and chronic kidney disease (CKD). The presence of these disorders is linked to a more severe course of COVID-19. Given the “epidemic” of obesity worldwide and the importance of obesity in the progression of COVID-19, we investigated the mechanisms through which obesity increases the susceptibility to and severity of COVID-19 to support the selection of more appropriate therapies for individuals with obesity.

## Introduction

With the acceleration of the coronavirus disease 2019 (COVID-19) pandemic worldwide, people are concerned about implementing the necessary measures to avoid infection and facilitate recovery should they become infected. Therefore, it is necessary to identify risk factors contracting COVID-19 and progressing to severe disease. Accumulating statistical analyses have shown that there is a higher risk of severe disease in individuals with obesity. An academic health system in New York reported that among 4,103 COVID-19 patients, a BMI>40 kg/m^2^ was the second strongest independent predictor of hospitalization after advanced age (1). It is worth noting that obesity is the chronic disease most closely related to critical illness, and the odds ratio for critical illness is higher among obese individuals than those with any cardiovascular or pulmonary disease. Most importantly, recent data from the UK, the USA, and Mexico revealed elevated mortality rates in COVID-19 patients with obesity (2–4). Considering that over one-third of the population worldwide is overweight or obese (5), it is crucial to discuss the mechanisms that make individuals with obesity more susceptible to severe disease.

Studies have shown that the binding of the spike protein(s) with angiotensin-converting enzyme 2 (ACE2) mediates the entry of severe acute respiratory syndrome coronavirus 2 (SARS-CoV-2) into host cells. The activation of the spike protein(s) depends on the activation of a serine protease in host cells (6). First, SARS-CoV-2 binds the N-terminal portion of the S1 protein unit to the ACE2 receptor pocket. Second, transmembrane protease serine 2 (TMPRSS2) and furin mediate protein cleavage between the S1 and S2 units, and the remaining viral S2 unit undergoes conformational rearrangement. Finally, SARS-CoV-2 fuses with the cellular membrane and enters the host.

**Figure 1:**
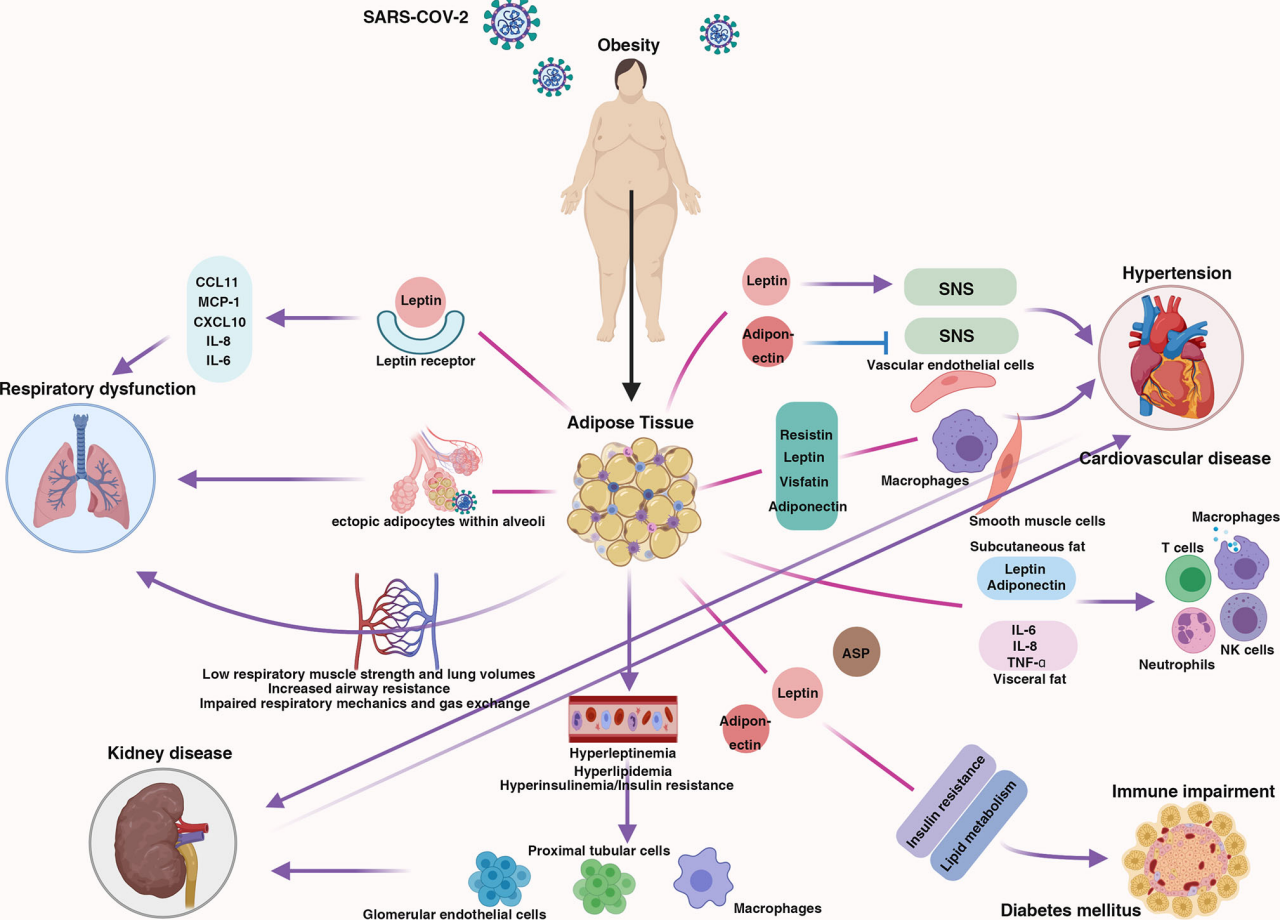
The centrality of obesity in the course of severe COVID-19. Schematic demonstrating the interaction between obesity and multiple body systems, contributing to severe COVID-19.

Obesity has a comprehensive effect on the human body. Obesity contributes to respiratory dysfunction and immune impairment and is also responsible for comorbidities related to severe COVID-19 (hypertension, cardiovascular disease, diabetes mellitus, and kidney disease) (**Figure 1**); thus, individuals with obesity may be more likely to develop severe COVID-19. Here, we discuss the mechanism by which obesity increases the likelihood of progression to severe COVID-19 and potential therapeutic strategies for obese individuals with severe COVID-19.

## Potential Mechanisms by Which Obesity Increases the Likelihood of Progression to Severe COVID-19

### Obesity and COVID-19

In this section, the mechanistic links between obesity and severe COVID-19 are discussed from the perspective of the pathophysiology of COVID-19, the immune system, and respiratory physiology.

ACE2 is the putative receptor mediating the entry of SARS-CoV-2 into host cells; therefore, ACE2 expression is closely correlated with susceptibility to COVID-19. In addition to the heart, vessels, gut, lung, kidney, testis, and brain, ACE2 is also expressed in adipose tissue (7, 8). Individuals with obesity have more adipocytes to express ACE2, making them more susceptible to contracting COVID-19. This finding is supported by a study revealing that elevated serum ACE2 levels in obese individuals are correlated with severe outcomes of COVID-19 (9). Moreover, a previous study suggested that furin expression is significantly enhanced during adipogenesis (10), and furin facilitates the entry of SARS-CoV-2 into cells and the release of virus particles from cells (11). Unfortunately, data regarding furin expression in patients with obesity and COVID-19 are currently lacking. Following their entry into host cells, viruses exploit the cellular translation apparatus to express viral proteins (12). Most coronaviruses are thought to undergo cap-dependent translation using eIF4F because of the 5′ cap structures in their genomic and subgenomic mRNAs (12). mTORC1 regulates eIF4F complex assembly and cap-dependent mRNA translation machinery by sensing nutrient availability (13). Obesity induces mTOR hyperactivation in multiple tissues (14), facilitating the replication of coronaviruses (15). The accelerated replication of SARS-CoV-2 mRNA also potentially increases susceptibility to severe COVID-19.

Adipose tissue is recognized as an endocrine organ. Different adipocytokine profiles are produced by subcutaneous and visceral fat, and adipose tissue plays an important role in the immune system. Subcutaneous fat produces higher levels of leptin and adiponectin (16). Leptin acts on monocytes/macrophages (17, 18) and induces neutrophil chemotaxis and oxygen radical release (19). Leptin has also been shown to modulate NK cell development and activation *in vitro* and *in vivo* (20, 21). Moreover, leptin deficiency leads to reduced levels of TNF-α and IL-18 and reduced T cell counts (22). Adiponectin is the only adipocytokine negatively related to body fat. Adiponectin induces the production of IL-10 and IL-1 receptor antagonists and reduces the production of TNF-α and IL-6 (23–25). The hypertrophy and hyperplasia of adipocytes in obese individuals disrupt the balance in adipokine production by inducing the secretion of proinflammatory adipokines, such as leptin, and decreasing the production of anti-inflammatory adipokines, especially adiponectin (26). Visceral fat produces relatively more proinflammatory cytokines, including IL-6, TNF-α, and IL-8 (16). Cytokine storm syndrome, which is triggered by IL-6, is the main cause of COVID-19-related death (27). Therefore, visceral fat is the more harmful of the two types of fat for patients with COVID-19. A research team from Sapienza University of Rome reported that among 150 patients with COVID-19, visceral fat was the strongest predictor of intubation, whereas an elevated subcutaneous fat area did not increase the risk of ICU admission (28). Another study also showed that for every increase of ten square centimeters in visceral fat area, the likelihood of ICU admission and mechanical ventilation increased 1.37- and 1.32-fold, respectively (29).

From a physiological perspective, obesity impairs respiratory mechanics and gas exchange, increases airway resistance, and lowers respiratory muscle strength and lung volumes (30). A study involving 121,965 patients showed that abdominal obesity was the strongest predictor of lung function impairment among the specific components of metabolic syndrome, with odds ratios (ORs) for impaired FEV (1) and FVC of 1.94 and 2.11, respectively (31). In addition, hypertrophic adipocytes produce high levels of adipokines, such as leptin, that bind to their receptors on airway epithelial cells, contributing to airway remodeling and hyperreactivity (32). These adipokines are also associated with airway inflammation. Leptin acts on lung fibroblasts and contributes to lung inflammation by inducing proinflammatory cytokines and chemokines (33). Initial findings suggest that high leptin levels are associated with more severe pulmonary inflammation in COVID-19 patients (34). Recent evidence has confirmed that the accumulation of lung parenchyma adipose tissue in subjects with obesity may contribute to the development of inflammatory infiltrate (35). It is noteworthy that intrapulmonary adipocytes can have an additional proinflammatory effect on the respiratory function of patients with COVID-19 (36). Excess fat can lead to the location of ectopic adipocytes within the alveolar interstitial space. Those adipocytes may be directly infected by SARS-CoV-2, leading to aggravation of the inflammatory infiltrate and massive interstitial edema. A meta-analysis that pooled three studies with 208 patients showed that patients who needed invasive mechanical ventilation (IMV) had higher visceral fat area values than patients who did not need IMV (37), indicating the risk of respiratory dysfunction in patients with obesity and severe COVID-19.

### Obesity-Associated Comorbidities and COVID-19

#### Cardiovascular and Metabolic Complications

A study published in The Lancet showed that approximately 1/3-1/2 of 41 COVID-19 patients had underlying diseases (13 [32%]), including diabetes (eight [20%]), hypertension (six [15%]), and cardiovascular disease (six [15%]) (38). Another analysis of 1527 patients showed that the prevalences of hypertension, cardio-cerebrovascular diseases, and diabetes were approximately two-, three-, and two-fold higher, respectively, in patients treated in the ICU or with severe COVID-19 than in their counterparts (39). These results showed that there is a relationship between cardiovascular and metabolic diseases and COVID-19 and its severity. Obesity is associated with adipose tissue dysfunction, which can lead to the onset of several pathologies, including hypertension, cardiovascular disease (CVD), and type 2 diabetes.

Activation of the renin-angiotensin system (RAS) and activation of the sympathetic nervous system (SNS) have been unequivocally associated with the development and persistence of hypertension. Adipose tissue not only contains most components of the RAS system (angiotensinogen, angiotensin-converting enzyme, angiotensin II and angiotensin II receptors) (40) but also secretes adipocytokines that participate in the regulation of SNS. For example, leptin increases sympathetic activity through a melanocortin-dependent pathway within the hypothalamus (41). In addition, adiponectin blocks the activation of the SNS by increasing endothelial NO synthase activity (42). Obesity also causes structural alterations in the kidneys and the loss of nephron function, which further elevates blood pressure (43).

Several studies have shown that obesity is linked to the risk of incident CVD (44, 45). The mechanism by which obesity affects the development of CVD is complex. In addition to well-known obesity-related factors (including hypertension, IR, and dyslipidemia), adipokines also seem to have adverse effects that promote CVD. Adipocytes secrete many proinflammatory cytokines, chemokines, and hormone-like factors, inducing the increased secretion of very-low-density lipoprotein, apolipoprotein B (apo B), and triglycerides in the liver (46). These liver-released molecules regulate the gene expression and function of vascular endothelial cells, arterial smooth muscle, and macrophages, exerting atherogenic effects on vessel walls (44, 45). Among the adipokines, resistin stimulates endothelial cells to express adhesion molecules, proinflammatory cytokines, and pentraxin, accelerating the development of coronary atherosclerosis (47). Leptin induces endothelial cell oxidative stress, increases the production of adhesion molecules and chemokines and promotes smooth muscle cell migration (44). Visfatin, localized to foam cell macrophages within unstable atherosclerotic lesions, has a key role in plaque destabilization (48). Additionally, elevated levels of visfatin cause vascular endothelial dysfunction (49). Adiponectin reduces the risk of coronary atherosclerosis by downregulating the expression of adhesion molecules on endothelial cells (50), decreasing endothelial oxidative stress and increasing eNOS activity (51). Recently, a case report showed the induction of endothelial inflammation in a patient with obesity (52), which confirms the role of obesity in endothelial dysfunction in patients with COVID-19. Importantly, vascular endothelial cells highly express ACE2, TMPRSS2, and furin (53), which makes the endothelium of patients with obesity readily accessible by SARS-CoV-2.

Type 2 diabetes is mainly characterized by lipid metabolic disorder and insulin resistance. The absence or excess of adipokines secreted by adipose tissue leads to severe alterations in lipid metabolism and insulin sensitivity. Studies have shown the molecular mechanisms by which adipokines modulate lipid metabolism and insulin resistance. For example, leptin blocks carbohydrate conversion into long-chain fatty acids (54) and promotes hormone-sensitive lipase expression but inhibits fatty acid synthase expression (55). In addition, leptin directly inhibits insulin binding by adipocytes, which contributes to insulin resistance (56). Adiponectin increases fatty acid oxidation and reduces glucose synthesis in the liver (57). It benefits insulin sensitivity *via* two receptors, ADIPOR1 and ADIPOR2 (58). The simultaneous disruption of both ADIPOR1 and R2 abolishes adiponectin binding and activity, leading to insulin resistance and glucose intolerance (59). Acylation-stimulating protein (ASP), a complement-derived adipokine, enhances insulin sensitivity by increasing glucose transport into adipocytes and blocks lipolysis by inhibiting hormone-sensitive lipase (60).

#### Kidney Disease

Renal impairment is a common phenomenon among COVID-19 patients. A retrospective study showed that 60%, 31%, 22%, 20%, 70%, and 96% of COVID-19 patients had proteinuria; elevated levels of BUN, SCr, uric acid (UA), D-dimer (DD); and radiographic abnormalities of the kidney, respectively (61). Furthermore, the prevalence of kidney disease on admission and the incidence of AKI during hospitalization are associated with in-hospital mortality (62). SARS-CoV-2 initiates and aggravates kidney damage, and patients with pre-existing chronic kidney disease (CKD) are more likely to develop severe COVID-19. A meta-analysis showed that COVID-19 patients with CKD have a 2.22-fold increased risk of developing severe disease (63). Previous studies have shown that weight loss facilitates a reduction in proteinuria (64), and body mass index (BMI) is a predictor of end-stage renal disease (ESRD) (65).

The CKD-related mechanisms induced by obesity include renal remodeling, hyperinsulinemia/insulin resistance, hyperleptinemia, hyperlipidemia, and essential hypertension. In the early stages of obesity, mesangial and capillary endothelial cells proliferate, the deposition of hyaluronate accelerates in the inner medulla, Bowman’s capsule space area is augmented, the glomerular basement thickens, and the focal formation of TGF-β increases (66–68). These changes in renal structure and function can lead to obstructed urine outflow and increased intrarenal pressure, which can induce renal impairment. Insulin resistance leads to higher glomerular pressure and albuminuria by increasing salt sensitivity (69). Consequently, insulin-sensitizing agents can attenuate proteinuria and renal injury (70). Hyperleptinemia has proliferative and sclerotic effects on glomeruli, stimulating glomerular endothelial cell proliferation and increasing TGF-β synthesis and collagen type IV production (71). Hyperlipidemia acts on proximal tubular cells, promotes monocyte/macrophage infiltration into glomeruli, and aggravates tubulointerstitial cells (72). Obesity causes increased renal sodium levels and impaired pressure natriuresis in patients with essential hypertension by activating the RAS and SNS and by altering intrarenal physical forces (43).

## Potential Therapeutic Strategies for Patients With Obesity

At present, no specific drugs are available that have been approved as treatments for COVID-19. With the increasing understanding of the pathophysiology of COVID-19, several strategies have been developed to improve the prognosis of patients with COVID-19. These strategies have been proposed based on the confirmation of their effectiveness in clinical studies and their theoretical rationality supported by scientific evidence. Their long-term efficacy needs to be observed over time.

## Pharmacological Interventions

Glucocorticoids have potent anti-inflammatory and antifibrotic properties, which may attenuate inflammation-mediated lung injury. Low doses of glucocorticoids facilitate the resolution of pulmonary and systemic inflammation in pneumonia by inhibiting proinflammatory cytokine production and the consequent cytokine storms (73). However, the use of glucocorticoids also leads to immunosuppression, slower pathogen clearance and accelerated virus replication (74). Therefore, it might be reasonable to administer low-dose glucocorticoids in the short term to patients with severe COVID-19 who have progressed to ARDS. A multicenter, randomized controlled trial demonstrated that dexamethasone significantly increased the survival duration and duration of freedom from mechanical ventilation during the first 28 days in COVID-19 patients with ARDS (75). Additionally, the administration of dexamethasone contributed to a lower 28-day mortality among patients undergoing IMV (29.3% vs. 41.4%) and among those receiving oxygen without IMV (23.3% vs. 26.2%) but not among those who were not receiving respiratory support (17.8% vs. 14.0%) (76). However, no analyses have evaluated the usefulness of dexamethasone in subpopulations of patients with COVID-19. In addition to anti-inflammatory, antifibrotic and immunosuppressive effects, glucocorticoids can impair glucose metabolism, leading to glucose homeostasis imbalance and hyperglycemia (77). Poorly controlled blood glucose is correlated with worse outcomes in patients with COVID-19 (78). Therefore, for obese individuals with severe COVID-19, the rational use of glucocorticoids may yield a better prognosis after cautiously weighing the benefits and risks.

Metabolic disorders and immune impairment, which are two important risk factors for COVID-19, have been found in obese patients. Therefore, overcoming oxidative stress and attenuating the inflammatory response may improve the outcome in these patients. Melatonin exerts an anti-obesity effect by reducing fat depot mass, inhibiting adipocyte hypertrophy, and lowering the levels of total cholesterol, triglycerides and LDL cholesterol. In addition, melatonin can reverse the obesity-induced overexpression of inflammatory cytokines in epididymal adipose depots, including leptin, IL-6, MCP-1, and TNF-α (79). An observational study involving 26,779 individuals found that the use of melatonin is significantly associated with a 28% reduced likelihood of a positive laboratory test result for SARS-CoV-2 (80). Additionally, melatonin administration after intubation contributed to a positive outcome in COVID-19 patients (81). Melatonin may serve as a potential therapeutic adjuvant to improve clinical outcomes in obese individuals with COVID-19.

## Stem Cell-Based Therapy

Mesenchymal stem cells (MSCs) can intrinsically express interferon-stimulated genes against viral infection (82). In particular, the interferon-induced transmembrane family (IFITM) prevents infection with some pathogenic viruses, including SARS-CoV (83). The accumulation of some MSCs in the lungs can suppress lung inflammation, protect alveolar epithelial cells, prevent pulmonary fibrosis, and improve lung function (84). The gene expression profile showed that MSCs are ACE2- and TMPRSS2-negative, indicating that MSCs cannot be infected by SARS-CoV-2 (85). The restoration of MSCs could alleviate SARS-CoV-2-induced immune dysfunction and tissue damage. A study involving 41 patients showed that the administration of MSCs has favorable effects in COVID-19 patients, including a faster time for the oxygenation index and lymphocyte count to return to within the normal range and reduced lung inflammation (86). A randomized controlled trial conducted by the University of Miami Miller School of Medicine found that umbilical cord mesenchymal stem cell (UC-MSC) infusions can increase patient survival to more than double (91% vs 42%) in subjects with COVID-19-related ARDS without serious adverse events (87). Obesity-associated factors can disrupt the functions of MSCs with regard to tissue regeneration, anti-inflammation, and immune modulation (88). Given the dysfunction of MSCs in obesity, stem cell-based therapy may be effective in these patients.

## Nonpharmacological Interventions

In addition to pharmacological interventions, diet therapy may be considered for patients with COVID-19, such as a eucaloric ketogenic diet (EKD). There is a growing interest in the clinical use of ketogenic diets, particularly in the context of severe obesity with related metabolic complications (89), in consideration of their role in the effective reduction of subjects at risk of SARS-COV-2 infection and worse outcome of disease. An EKD can serve as a metabolic treatment for cytokine storm syndrome by reducing aerobic glycolysis in activated M1 macrophages, thereby limiting their proinflammatory functions (90). Meanwhile, since M2 macrophages predominantly express OXPHOS enzymes, the supply of free fatty acids from an EKD facilitates the metabolism of anti-inflammatory M2 macrophages and counteracts proinflammatory cytokines in the alveolar space by producing anti-inflammatory cytokines (IL-10 and IL-1) (91, 92). In addition, the finding that excessive lactate inhibits IFN-I production indicated that the inhibitory effects of an EKD on glucose metabolism and lactate production can promote IFN-I production, reducing the likelihood of virus infection (93). Recently, an *in vitro* study showed that targeting glycolysis with a deoxy-D-glucose glycolysis inhibitor inhibited the replication of SARS-CoV-2 in cells (94). The similar antiglycolytic effect obtained by means of KDs may make an EKD a therapeutic strategy for COVID-19. A high-fat, low-carbohydrate diet is also beneficial for patients with respiratory failure or ARDS. Studies found that a high-fat, low-carbohydrate diet shortened the duration of ventilator use in patients with respiratory failure and ARDS (95, 96). Therefore, an EKD together with moderate high-fat supplementation may blunt the COVID-19-induced cytokine storm. A clinical study is warranted to observe the outcome in COVID-19 patients fed a high-fat EKD diet.

Vitamin D deficiency increases the risk of various chronic illnesses, including diabetes mellitus, hypertension, chronic cardiovascular and respiratory diseases, and cancers (97), all of which are linked to the progression of COVID-19. Recently, low 25-hydroxyvitamin D levels have been identified as being related to susceptibility to infection with SARS-CoV-2 (98) and progression to severe COVID-19 (99). In vivo experiments suggested that vitamin D has protective effects against ARDS by blocking AngII expression (100). Notably, the level of plasma AngII in COVID-19 patients is significantly increased and linearly related to the viral load and lung injury (101). Therefore, vitamin D supplementation may prevent adverse COVID-19 outcomes. Physically, vitamin D stabilizes the pulmonary epithelial barrier to prevent virus infection (102) and stimulates epithelial repair to attenuate lung injury (103). At the cellular level, vitamin D can inhibit inflammatory processes to prevent ARDS by reducing the expression of proinflammatory cytokines by T helper type 1 (Th1) cells and increasing the production of anti-inflammatory cytokines by macrophages (104). A clinical study involving 76 patients with COVID-19 demonstrated that 25-hydroxyvitamin D administration significantly reduced the need for admission to the ICU (105). Given the high prevalence of vitamin D deficiency among people with obesity (106), it may be important to provide these patients with supplemental vitamin D when they have COVID-19.

Previous studies found that individuals participating in regular exercise have lower incidence and mortality rates for influenza and pneumonia than inactive individuals (107, 108). Exercise interventions may be beneficial for obese patients with COVID-19. Viral infections, including infections with SARS-CoV-2, can accelerate endothelial dysfunction, with reduced nitric oxide (NO) expression and abnormal coagulation (109). Coagulation abnormalities have been reported to be associated with a poor prognosis in patients with COVID-19 (110). The restoration of NO expression induced by physical activity can contribute to pulmonary vasodilation and antithrombotic activity (109), thus lowering the risk of disseminated intravascular coagulation (DIC) in patients with severe COVID-19. Regular moderate exercise may help reduce systemic inflammation by inhibiting the TLR-inflammatory signaling pathway (111). Physical activity-mediated AMPK signaling induces the phosphorylation of ACE2 and the subsequent conversion of Ang II to Ang 1-7 (112), thus reducing lung inflammation. In addition, regular moderate exercise can create an anti-inflammatory environment in muscle and adipose tissue through the involvement of macrophages, cytokines and adipokines (113). Physical inactivity is very common among people with obesity; therefore, regular moderate exercise can be included as part of their rehabilitation after COVID-19.

## Conclusions

In conclusion, obesity plays a comprehensive role in the course of severe COVID-19. Individuals with obesity need more nursing care during COVID-19 and rehabilitation support as they recover. In addition to the evaluation of standard hospital parameters (such as the Sequential Organ Failure Assessment and the levels of D-dimer and proinflammatory markers), the measurements associated with obesity, such as anthropometric and metabolic parameters, need to be seriously considered to better estimate patient risk and to aid in the selection of more appropriate therapies. It is worth noting that weight loss may be beneficial for individuals with obesity as a precautionary measure and to assist in the rehabilitation or treatment of patients with COVID-19. A variety of appropriate treatments for COVID-19 patients with obesity could be selected to obtain better curative effects.

## Author Contributions

DL, TZ, and YW performed the literature search and manuscript drafting. LX supervised and revised the manuscript. All authors contributed to the article and approved the submitted version.

## Funding

Funding was obtained from National Key Research and Development Program of China (no. 2018YFC1312103, LX) and National Natural Science Foundation of China (nos. 81972237, 81772623, LX).

## Conflict of Interest

The authors declare that the research was conducted in the absence of any commercial or financial relationships that could be construed as a potential conflict of interest.
